# Impact of Evidence-Based Quality Improvement on Tailoring VA’s Patient-Centered Medical Home Model to Women Veterans’ Needs

**DOI:** 10.1007/s11606-024-08647-4

**Published:** 2024-02-29

**Authors:** Elizabeth M. Yano, Claire Than, Julian Brunner, Ismelda A. Canelo, Lisa S. Meredith, Lisa V. Rubenstein, Alison B. Hamilton

**Affiliations:** 1 VA Los Angeles HSR&D Center for the Study of Healthcare Innovation, Implementation and Policy, VA Greater Los Angeles Healthcare System, 16111 Plummer Street (Mailcode 152), Sepulveda, CA 91343 USA; 2 Department of Health Policy and Management, UCLA Fielding School of Public Health, 650 Charles E. Young Drive South, Los Angeles, CA 90095 USA; 3 Department of Medicine, UCLA Geffen School of Medicine, 855 Tiverton Drive, Los Angeles, CA 90024 USA; 4 National Precision Oncology Program, Veterans Health Administration, Washington, DC USA; 5 RAND Corporation, 1776 Main Street, Santa Monica, CA 90401-3208 USA; 6 Department of Psychiatry and Biobehavioral Sciences, UCLA Geffen School of Medicine, 757 Westwood Plaza, Los Angeles, CA 90095 USA

**Keywords:** primary care, patient-centered medical home, women’s health, women Veterans, VA healthcare system.

## Abstract

**Background:**

Women Veterans’ numerical minority, high rates of military sexual trauma, and gender-specific healthcare needs have complicated implementation of comprehensive primary care (PC) under VA’s patient-centered medical home model, Patient Aligned Care Teams (PACT).

**Objective:**

We deployed an evidence-based quality improvement (EBQI) approach to tailor PACT to meet women Veterans’ needs and studied its effects on women’s health (WH) care readiness, team-based care, and burnout.

**Design:**

We evaluated EBQI effectiveness in a cluster randomized trial with unbalanced random allocation of 12 VAMCs (8 EBQI vs. 4 control). Clinicians/staff completed web-based surveys at baseline (2014) and 24 months (2016). We adjusted for individual-level covariates (e.g., years at VA) and weighted for non-response in difference-in-difference analyses for readiness and team-based care overall and by teamlet type (mixed-gender PC-PACTs vs. women-only WH-PACTs), as well as post-only burnout comparisons.

**Participants:**

We surveyed all clinicians/staff in general PC and WH clinics.

**Intervention:**

EBQI involved structured engagement of multilevel, multidisciplinary stakeholders at network, VAMC, and clinic levels toward network-specific QI roadmaps. The research team provided QI training, formative feedback, and external practice facilitation, and support for cross-site collaboration calls to VAMC-level QI teams, which developed roadmap-linked projects adapted to local contexts.

**Main Measures:**

WH care readiness (confidence providing WH care, self-efficacy implementing PACT for women, barriers to providing care for women, gender sensitivity); team-based care (change-readiness, communication, decision-making, PACT-related QI, functioning); burnout.

**Key Results:**

Overall, EBQI had mixed effects which varied substantively by type of PACT. In PC-PACTs, EBQI increased self-efficacy implementing PACT for women and gender sensitivity, even as it lowered confidence. In contrast, in WH-PACTs, EBQI improved change-readiness, team-based communication, and functioning, and was associated with lower burnout.

**Conclusions:**

EBQI effectiveness varied, with WH-PACTs experiencing broader benefits and PC-PACTs improving basic WH care readiness. Lower confidence delivering WH care by PC-PACT members warrants further study.

**Trial Registration:**

The data in this paper represent results from a cluster randomized controlled trial registered in ClinicalTrials.gov (NCT02039856).

**Supplementary Information:**

The online version contains supplementary material available at 10.1007/s11606-024-08647-4.

## INTRODUCTION

Women Veterans are a numerical minority in the Veterans Health Administration (VA), chiefly reflecting historical differences in the composition of the military and thus the population of US Veterans upon military discharge. However, women are now the fastest growing segment of new VA users, with their own set of complex healthcare needs, including higher rates of service-connected disability and military sexual trauma (MST) in addition to gender-specific care needs that require gender-sensitive, trauma-informed care.^1^ As a result, VA has prioritized delivery of comprehensive women’s health (WH) services through gender-specific care models (e.g., WH clinics, designated WH providers) to meet their needs and mitigate gender disparities.^2–4^

In 2010, VA mounted efforts to nationally implement patient-centered medical homes, called Patient Aligned Care Teams (PACT),^5^ while parallel policy directives guided field-based requirements for delivering comprehensive PC for women Veterans.^6^ PACT delivers care to assigned patient panels through teamlets (provider, nurse, medical assistant, clerk), which are supported by a larger team (e.g., social workers, health coaches, clinical pharmacists). Consistent with VA policy, VAMCs may deliver PC to women Veterans in mixed-gender panels by PACT teamlets in general PC clinics (PC-PACTs) (Model 1) or in female-only panels seen by teamlets led by a designated WH provider (WH-PACTs). WH-PACTs may be co-located in general primary care (Model 2) or in a separate WH clinic (Model 3).

Given that the majority of patients in VA were (and still are) men, many VA primary care providers (PCPs) were historically less up-to-date on WH care delivery, including gender-specific services, and lacked experience addressing the needs of women Veterans with MST histories, which is concerning given that over 60% of women who routinely use VA PC have such histories.^4,7^ Team function was also a concern as women’s clinics were not appropriately staffed for women’s preventive care (e.g., chaperone use for gender-sensitive exams such as Pap smears) and staff were frequently shared across multiple clinics.^8^ Higher rates of burnout among providers in WH clinics vs. general PC clinics also raised concerns about how well PACT was working given the extra resources and specialized knowledge needed to care for complex women Veteran patients.^9^

In response to these challenges, we partnered with the VA Office of Women’s Health (OWH) to test the effectiveness of an evidence-based quality improvement (EBQI) approach to tailoring PACT to better meet women Veterans’ needs.^10^ EBQI is an implementation strategy that emphasizes a clinical-research partnership approach through multilevel stakeholder engagement, QI training, formative data feedback, technical support, and practice facilitation.^11,12^ EBQI has been shown to support implementation of evidence-based practices, including smoking cessation guidelines, depression collaborative care, and PACT implementation.^13–17^ In this paper, we report results of our cluster randomized trial of EBQI on WH care readiness, team-based care, and burnout.

## METHODS

### Study Design and Setting

We evaluated effectiveness of EBQI in a parallel, two-arm cluster randomized controlled trial (cRCT) from 2014 to 2016, beginning approximately 4 years into PACT implementation.^10^ We randomly assigned 12 VA medical centers (VAMCs) to EBQI vs. routine PACT implementation in an unbalanced 2:1 ratio in each of 4 participating Veterans Integrated Service Networks (VISNs) (8 experimental vs. 4 control VAMCs). Participating VAMCs were in urban and rural locations spanning nine states.^10^ All sites were part of the VA WH Practice-Based Research Network (WH-PBRN).^18^

### Ethical Review

The study was approved by the VA Greater Los Angeles Healthcare System Institutional Review Board (IRB); VA’s Central IRB determined sites were not engaged in research, permitting review by the home institution IRB only. Provider/staff survey data were collected in collaboration with in collaboration with the RAND Corporation, with study procedures approved by their IRB as well, with a waiver of documentation of consent. We obtained required approvals from VA labor management/unions and the VA National Center for Organizational Development (NCOD); however, NCOD disapproved baseline collection of burnout data (given persistently high rates nationwide), but allowed measure inclusion at 24 months (resulting in post-only comparisons).

### EBQI Approach

**Figure 1:**
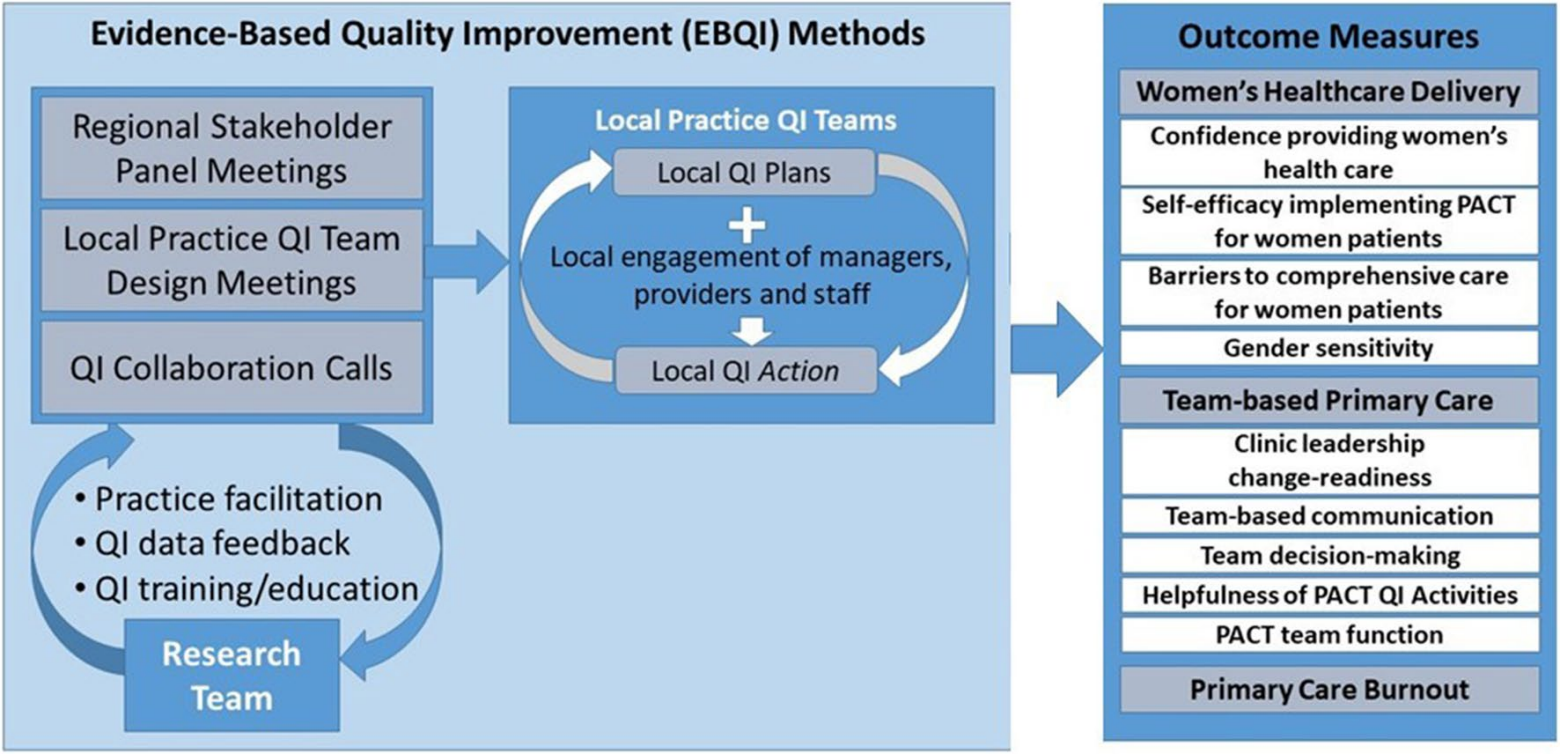
Conceptual model of evidence-based quality improvement effects on provider/staff outcomes.

We launched EBQI with in-person multilevel stakeholder meetings in each VISN (Fig. 1), using expert panel methods to come to consensus on QI priorities, summarized in roadmaps.^19^ We then trained two core members of each local team in EBQI and provided external practice facilitation over 24 months to help them use QI tools (e.g., process maps, measures) to conduct projects chosen from their VISN-specific QI roadmaps. We provided ongoing technical support, coaching/mentoring, and use of data from patient and provider/staff surveys and key stakeholder interviews^19^ to inform plan-do-study-act cycles within their projects. We supported across-EBQI-site calls and within-VAMC/VISN leadership reporting to share progress, culminating in capstone VISN-specific stakeholder meetings.^10^

Control VAMCs received standard policy guidance on PACT implementation and provision of comprehensive women’s health services that were disseminated to all VA healthcare facilities nationwide.

### Sample

We surveyed all PC and WH-PACT teamlet members (PCPs, nurses, medical assistants, clerks) and extended PACT teams (e.g., social workers, pharmacists, health coaches) in participating VAMCs (September 2014–October 2016). We identified eligible PACT personnel using the VA Support Service Center (VSSC) Primary Care Reports for each geographically distinct station number and code. We complemented VSSC lists with VA Corporate Data Warehouse files to identify additional teamlets and team members not found in VSSC, obtaining staff identifiers, full vs. parttime status, gender, position title, and panel size. We excluded PACTs for special populations (e.g., spinal cord injury), resident-only teamlets, and home-based primary care.

### Data Collection Procedures

At baseline and 24 months, providers and staff were contacted by email and provided with a brief study summary, answers to frequently asked questions, and a site-specific endorsement letter from a local leader, followed within 1–2 weeks by an email with a person-specific survey weblink and email-based follow-up of non-responders.

### Measures

#### Characteristics of Participating VAMCs

We obtained urban/rural location from the VA Site Tracking database, which defines location based on the US Census. We determined academic affiliation using data validated by the VA Office of Academic Affiliations on medical school affiliations for each VAMC. Facility complexity is a VA-generated 5-point ordinal composite scale of patient volume, risk, scope of practice, teaching/research funding, and level of intensive care (e.g., 1a = most complex). Volume of women Veterans and total Veterans served were obtained from VSSC’s Primary Care Almanac.

#### Dependent Variables

Table 1 provides details on outcome measures, including Cronbach’s alpha coefficients for each scale. Measures included (1) WH care readiness (i.e., confidence providing WH care [clinicians only], self-efficacy implementing PACT for women, barriers to providing comprehensive care to women, gender sensitivity); (2) team-based care (i.e., clinic leadership change-readiness, team communication, decision-making, PACT-related QI activities, team functioning); and (3) PC burnout (24-month wave only due to VA oversight issues noted above). Measure scales and items are in Appendix 1.

**Table 1:** Clinician/Staff Survey Measures*

Measure	Description
Women’s health care readiness	
Confidence providing women’s health care	10 items rating confidence in ability to provide each of the following services for women patients at their VA (e.g., conducting well-woman exams; contraception counseling; evaluation and/or management of acute/chronic pelvic pain or menopause management; screening for military sexual trauma, etc.) on a 4-point Likert scale (1 = not at all confident to 4 = very confident) (Cronbach’s *α* = 0.93)
Self-efficacy/readiness for implementing PACT for women	6 items adapted from the change efficacy subscale of a readiness for organizational change scale^38^ to relate to VA women’s primary care, rating statements about implementation of PACT for women patients (e.g., “As we implement PACT for women patients, I feel I can handle my role with ease.”) on a 5-point Likert agreement scale (reverse coded negatively words items and averaged into single composite score) (Cronbach’s *α* = 0.81)
Barriers to providing comprehensive care for women patients	15 items rating factors that could limit ability to provide comprehensive primary care for women patients at patient-, provider, and system-levels (e.g., inadequate training, inadequate space/structure, limited female staff to serve as chaperones) on a 3-point scale (1 = does not limit, 2 = limits somewhat, 3 = limits a great deal), adapted from a general primary care survey^39^ (Cronbach’s alpha = 0.91)
Clinician/staff gender sensitivity^2^	10 items adapted from 29-item Gender Sensitivity subscale of Gender Awareness Inventory^28^ (e.g., “Sometimes I wish VA primary care clinics had only male patients,” “Special women’s clinics should be at all VA health care facilities.”) rated on a 5-point Likert agreement scale (reverse coded negatively worded items and averaged into single composite score) (Cronbach’s *α* = 0.80)
Team-based care	
Clinic leadership change-readiness	6 items adapted from the leadership subscale from the Organizational Readiness for Change survey^40^ (e.g., “Encourages and supports changes in clinic patterns to improve patient care.” “Recognizes and rewards progress in implementing change with our clinic.”) rated on a 5-point Likert agreement scale (Cronbach’s *α* = 0.93)
Team-based communication	5 items, with 3 items adapted from the Survey of Organizational Attributes for Primary Care (SOAPC)^41^ and 2 new items specific to VA teamlets rating how team members communicate across disciplines (e.g., “Co-workers from different backgrounds frequently interact to solve problems.”) on a 5-point Likert agreement scale (Cronbach’s *α* = 0.79)
Decision-making	6 items adapted from the Survey of Organizational Attributes for Primary Care (SOAPC),^41^ rating agreement with statements about participation in decision-making (e.g., “Staff and clinicians are involved in developing plans for improving quality.”) on a 5-point Likert agreement scale (Cronbach’s alpha = 0.86)
PACT QI activities exposure and helpfulness	5 items adapted from a VA general primary care PACT survey, asking whether respondent had been exposed to each activity (e.g., performance feedback reports, involved in small tests of change to improve quality) and if yes, how helpful the activity was on a 3-point Likert scale from 1 = not at all helpful to 3 = very helpful (Cronbach’s *α* = 0.82)
PACT team function	4 items adapted from the Team Diagnostic Survey (TDS)^42^ integrating concepts of team knowledge, skills, and group process to PACT language (e.g., “Members of our PACT teamlet for women patients actively share their special knowledge and expertise with one another.”) rated on a 5-point Likert agreement scale (Cronbach’s *α* = 0.88)
Primary care burnout	
Clinician/staff burnout†	Single item rating of statement, “I feel burned out from my work” (7-point scale from “never” to “every day”)^43^ from Maslach Burnout Inventory^44^ (dichotomized as yes = every week to every day and no = never to less than a few times a month

^*^All measures were included in surveys at baseline and 24-month follow-up, with the exception of burnout, which was included in the 24-month wave only because the study team was not allowed to include a baseline measure by a national VA group overseeing any clinician/staff surveys that spanned multiple VISNs (rationale was that VA primary care burnout was already so high nationally that further measurement was not necessary)

All Cronbach’s alpha coefficients are based on the adapted/shortened measures used in the trial and were generated from these trial survey data

#### Covariate Measures

We measured individual-level characteristics including provider type (staff vs. PCP), gender (female vs. male), age group (≥ 50 years vs. < 50), race (non-White vs. White), fulltime (vs. parttime), experience in WH care (i.e., having cared for at least 50% women patients for 3+ years), and length of VA service (years). We also measured practice environments, including clinic urban/rural location and whether they worked in a WH-PACT (yes/no).

### Statistical Analysis

Baseline characteristics of clinicians/staff were compared by EBQI vs. control using *t*-tests for continuous variables and *Χ*^2^ tests for categorical variables. Variables hypothesized to be associated with each outcome measure were evaluated for collinearity and subsequently included in each regression model in an intent-to-treat analysis.^20,21^

We weighted clinician/staff data for likelihood of enrollment and drop-out (attrition at 24 months) using baseline measures of gender, marital status, years worked in the VA, VAMC, and position type (e.g., physician vs. nurse) to better represent the target population of all PCPs and staff. Final weights were the product of non-response and attrition weights. We used difference-in-differences (DID) analyses using generalized linear models, adjusting for survey weights, time (baseline vs. 24 months), EBQI (vs. control), gender, race/ethnicity, years worked in VA, WH-PACT (vs. PC-PACT), clinician (vs. staff), fulltime (vs. parttime) employment, and percent of women Veterans receiving care at their VA. Given different PACT teamlet options for delivering women’s primary care, we also examined EBQI effects by type of PACT teamlet (PC-PACTs [Model 1] vs. WH-PACTs [Models 2–3]). We used STATA 15.1 for all analyses.

## RESULTS

### Characteristics of Participating VAMCs, Providers, and Staff

**Figure 2:**
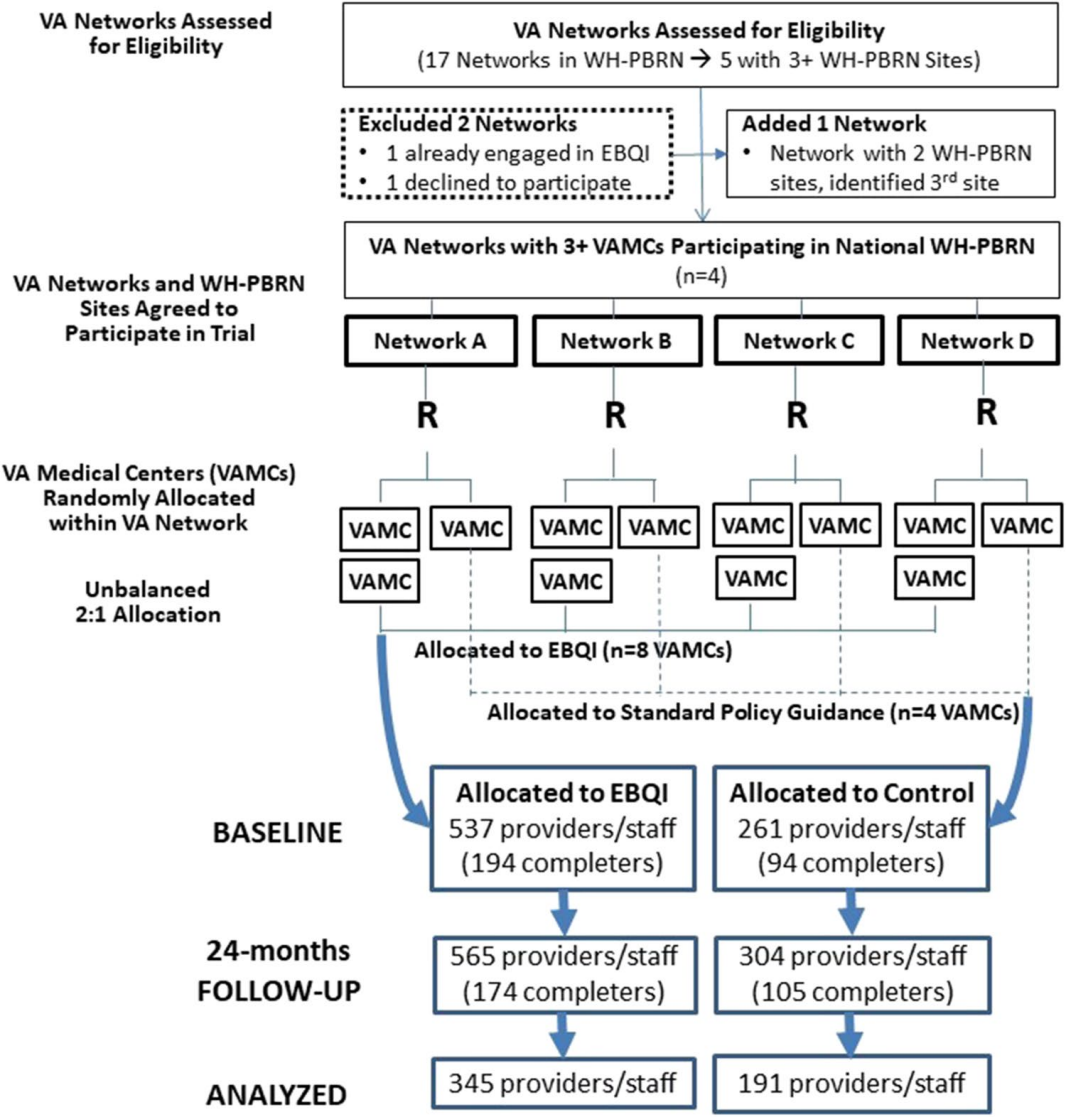
CONSORT flow diagram for the cluster randomized trial of evidence-based quality improvement (EBQI).

Participating VAMCs were predominantly in the Midwest (6) and Northeast (5) regions of the USA, with one site in the South (Table 2). All were academically affiliated, ranging in size (5040–35,285 Veteran users, 331–2622 women Veteran users, 2.3–13.2% women Veterans-to-total Veterans served in the prior year). All had mixed-gender PC clinics in addition to WH in PC or in a separate WH clinic, where a majority of women patients were seen (Tables 2 and 3). A higher percent of clinician/staff respondents were in WH-PACTs (45.8%) than PC-PACTs (33.5%) (Table 2). Response rates ranged from 30 to 36%. Figure 2 provides an overview of the CONSORT flow diagram of VISNs to VAMCs to clinicians/staff in this trial.

**Table 2:** Pre-implementation Characteristics of Participating VA Medical Centers and Clinicians/Staff

Multilevel characteristics at baseline	EBQI sites	Usual PACT control sites
Medical center characteristics	(*n* = 8)	(*n* = 4)
Urban location (vs. rural)	87.5%	75.0%
Regional distribution		
• Northeast	37.5%	50%
• Midwest	50.0%	50%
• South	12.5%	---
Academically affiliated	100%	100%
Mean # Veterans served (FY12)	17,156	16,652
Mean # women Veterans served (FY12)	1073	1310
Facility complexity*		
• High	75%	75%
• Medium	25%	---
• Low	---	25%
Women’s primary care arrangements^†^		
• Comprehensive women’s health clinic (Model 3)	62.5%	75.0%
• Separate women’s clinic in general primary care (Model 2)	25.0%	---
• Mixed-gender (integrated) primary care (Model 1)	100%	100%
Clinician/staff characteristics^‡^		
Number of providers and staff with completed surveys at baseline and 24-month follow-up	345	191
Percent female	255 (73.9%)	146 (76.4%)
Percent in a Women’s Health PACT (vs. PC-PACT)	158 (45.8%)	64 (33.5%)
Percent fulltime VA employee	312 (90.4%)	176 (92.2%)

^*^Facility complexity is a VA-generated weighted scale comprised of patient volume, patient risk (based on high-risk patient categories that generate greater resource allocation), scope of practice based on # of physician (MD) specialists, level of teaching and/or research funding, and level of intensive care unit (ICU) (e.g., levels 1–5). VA defines high complexity (1a, 1b, 1c) as those VAs with the largest patient volumes, patient risks, teaching and research, highest #s of MD specialists, and level 4–5 ICUs. Medium complexity VAs (2) have medium patient volume, patient risks, some teaching and/or research, and contain level 3 and 4 ICUs, while low complexity VAs (3) have the smallest levels of patient volume and risk, little or no teaching or research, the lowest # of physician specialists, and contain level 1 and 2 ICUs

Participating VAMCs were 1a and 1b (which we combined as high complexity, 2 (medium complexity), and 3 (low complexity)

^†^Sums to over 100% because all but 2 participating facilities offered more than one primary care arrangement for women Veterans

^‡^Clinician/staff characteristics for WH-PACTs vs. PC-PACTs, respectively: # with completed surveys at baseline and 24 months are 91 and 113; weighted % female clinicians/staff were 83.9% vs. 58.4% (***p*** < 0.001), and weighted % fulltime employee were 91.7% vs. 93.0% (***p*** = 0.60)

**Table 3:** Characteristics of EBQI Activities at Participating Sites

EBQI VAMC*	Local EBQI team composition†			% WVs seen in each type of PACT‡ (# of WH-PACT teamlets)			Focus of local QI projects
				Model 1	Model 2	Model 3	
	WHMD	WVPM	Other				
A1	X	X		3%	---	97% (7)	• ↑ team climate through virtual huddles • Cardiovascular disease (CVD) risk reduction
A2	X	X	X	20%	---	80% (7)	• ↑ team function (e.g., huddle checklist) • ↑ culture in VAMC (more welcoming, safe) • Training residents in trauma-sensitive care
B1	X	X	X	2%	98% (2)	---	• ↑ assignment of WVs to WH-PCPs • Improve teratogen prescribing practices
B2		X	X	33%	---	67% (1)	• ↑ assignment of WVs to WH-PCPs • ↑ labs drawn before PC appointment • ↑ outreach to WVs without phone to contact clinic • ↑ mental health access to WVs in crisis before 1^st^ PACT visit
C1	X	X		7%	---	93% (9)	• ↑ mammography screening rates • ↑ culture (↓ stranger harassment of WVs at VA
C2	X	X		1%	99% (4)	---	• ↑ coupled reporting of cervical cancer screening and HPV results • ↑ mammogram tracking and care coordination
D1	X	X	X	28%	---	72% (1)	• ↑ follow-up of cervical cancer screening results
D2		X	X	17%	83% (2)	---	• ↑ follow-up of breast cancer screening results • ↓ harassment of WVs at VA • ↑ environment safety through shared medical appointments

^*^The four participating VISNs are represented as A, B, C, and D, with the two VAMCs randomly assigned to EBQI in each VISN denoted as 1 and 2 within each VISN

^†^*WHMD*, Women’s Health Medical Director (in charge of women’s primary care or WH-PACT); *WVPM*, Women Veteran Program Manager (responsible for oversight of local women Veterans’ programs, one required at each VAMC nationwide). Other EBQI team members included a psychologist (A2), local WH-focused health services researcher (B1, D1), WH staff members (B1, B2, D2), and/or WH fellow (B1, D1)

^‡^Model 1 represents mixed gender general primary care clinics (PC PACT); Model 2 represents women’s clinics and/or WH-PACT teamlets organized within general primary care clinics; Model 3 represents comprehensive women’s health clinics in separate space

### EBQI Implementation

Multilevel panels in each VISN came to consensus on 7–10 priorities (e.g., improving gender-specific preventive practices) (roadmaps available on request) and collaboratively decided on key WH personnel (e.g., WH medical director) to lead each VAMC’s local QI team. Teams subsequently chose projects to focus on during the 24-month EBQI period (Table 3). Facilitation calls were arranged for every 2–8 weeks, depending on local QI team availability, over 24 months. Calls focused on developing process maps, selecting measures, identifying and helping teams engage local process “owners,” providing technical support, and creating brief action plans for both QI and research teams before the next call. Formative feedback was provided an average of five times across EBQI VAMCs, and included data from local and all-EBQI VAMC reports.

### Effectiveness of EBQI on Main Outcomes

Table 4 shows unadjusted comparisons of EBQI vs. control VAMC outcomes at baseline and 24 months. At baseline, control VAMCs had higher self-efficacy for implementing PACT for women (3.92 vs. 3.31, *p* = 0.027) and team functioning (4.10 vs. 3.70, *p* = 0.041) than EBQI VAMCs (Table 4). From baseline to 24 months, EBQI sites increased gender sensitivity, leadership change-readiness, team-based communication, decision-making, and PACT-related QI (all *p* < 0.05), and barriers decreased over time (*p* < 0.001). Control sites increased confidence delivering WH care, decision-making, and PACT-related QI (*p* < 0.05) and barriers decreased over time (*p* = 0.008). Unadjusted comparisons at 24 months revealed higher confidence in control vs. EBQI VAMCs (3.17 vs. 2.73, *p* = 0.013) (Table 4). Unadjusted 24-month burnout was not significantly different between EBQI and control VAMCs overall (19.2% vs. 24.4%, *p* = 0.38) or for PC-PACTs (22.4% vs. 21.0%, *p* = 0.88), but was marginally lower in WH-PACT EBQI sites (14% vs. 32%, *p* = 0.053) (not in table). (Appendix Table 2 shows unadjusted outcomes of PC-PACTs and WH-PACTs over time.)

**Table 4:** Unadjusted Comparisons of Outcome Measures Between EBQI and Control VAMCs

Outcome measures	Baseline			24-month follow-up		
	EBQI	Control	*p*-value	EBQI	Control	*p*-value
Women’s health care readiness						
Confidence in delivering WH care* (mean score on 4-point scale, 1 = not at all, 2 = somewhat, 3 = moderately, 4 = very confident)	2.86	2.53	0.18	2.73	3.17	0.013
Self-efficacy for implementing PACT for women (mean score on 5-point agreement scale, 1 = strongly disagree to 5 = strongly agree)	3.31	3.92	0.03	3.35	3.54	0.26
Barriers to providing comprehensive care for women patients (mean score 3-point scale, 1 = does not limit, 2 = limits somewhat, 3 = limits a great deal)	1.76	1.68	0.42	1.83	1.43	0.45
Gender sensitivity (mean score on 5-point agreement scale, 1 = strongly disagree to 5 = strongly agree)	4.04	4.15	0.24	4.20	4.14	0.52
Team-based care						
Clinic leadership change-readiness (mean score on 5-point agreement scale, 1 = strongly disagree to 5 = strongly agree)	3.26	3.31	0.74	3.54	3.49	0.74
Team communication (mean score on 5-point agreement scale, 1 = strongly disagree to 5 = strongly agree)	3.39	3.46	0.55	3.66	3.72	0.58
Team-based decision-making (mean score on 5-point agreement scale, 1 = strongly disagree to 5 = strongly agree)	3.46	3.42	0.76	3.67	3.80	0.27
Helpfulness of PACT QI activities (mean score on 3-point scale, 1 = not at all helpful, 2 = somewhat helpful, 3 = very helpful)	1.80	1.83	0.75	2.27	2.26	0.85
PACT team functioning (mean score on 5-point agreement scale, 1 = strongly disagree to 5 = strongly agree)	3.70	4.10	0.041	3.82	3.98	0.28

Negatively worded survey items were reverse coded; high scores reflect high attribute (e.g., high confidence, high barriers, high function). Weighted for non-response

^*^The items for this measure were asked among clinicians only and not staff

Table 5 shows multivariate results of EBQI effectiveness analyses, adjusting for individual-level covariates. Study-wide, EBQI was associated with lower confidence in providing WH care (DID, −0.68; 95% CI, −1.20, −0.16; *p* < .05), improved gender sensitivity (DID, 0.33; 95% CI, 0.10, 0.57; *p* < .01), and marginal improvements in self-efficacy and team function.

**Table 5:** Effects of EBQI on Outcome Measures Overall and by Type of PACT: Analysis of Difference in Differences Adjusted for Individual-Level Covariates^1^

Outcome measures	Overall	Type of PACT teamlet	
		PC-PACT	WH-PACT
	Mean difference-in-differences (95% confidence interval)		
Women’s health care readiness			
Confidence in providing women’s health^2^	−0.68 (−1.20, −0.16)*	−1.0 (−1.70, −0.26)**	−0.35 (−0.78, 0.07)
Self-efficacy/readiness for implementing PACT for women	0.57 (−0.08, 1.21)†	1.0 (0.01, 1.94)*	0.21 (−0.17, 0.60)
Barriers to providing comprehensive care for women patients	−0.08 (−0.31, 0.51)	0.09 (−0.23, 0.42)	−0.18 (−0.52, 0.16)
Gender sensitivity	0.33 (0.10, 0.57)**	0.35 (0.02, 0.70)*	0.20 (−0.13, 0.54)
Team-based care			
Leadership norms/readiness to change	0.28 (−0.15, 0.71)	−0.05 (−0.65, 0.55)	0.80 (0.26, 1.34)**
Communication within clinic	0.10 (−0.24, 0.44)	−0.30 (−0.71, 0.12)	0.60 (0.04, 1.17)*
Decision-making	0.01 (−0.34, 0.36)	−0.23 (−0.69, 0.23)	0.44 (−0.05, 0.93)
Helpfulness of PACT QI activities	0.09 (−0.16, 0.35)	0.11 (−0.24, 0.46)	0.09 (−0.28, 0.46)
PACT team function	0.43 (−0.01, 0.87)‡	−1.25 (−2.91, 0.41)	0.55 (0.10, 1.01)*

^*^p < 0.05; **p < 0.01; †*p* = 0.085, ‡*p* = 0.055

^1^Difference-in-difference analyses were adjusted for gender, race-ethnicity, years in VA, work primarily on general primary care vs. WH-PACT teamlet, provider vs. staff, full vs. parttime, and percent of women Veterans at their VA, and weighted for likelihood of enrollment and drop-out (attrition)

^2^Asked of clinicians only

Results for PC-PACT vs. WH-PACT teamlets demonstrated substantial variations in EBQI effects (Table 5). In PC-PACTs, EBQI was still associated with *lower* confidence in providing WH care (DID, −1.0; 95% CI [−1.70,−0.26]; *p* < 0.01), but greater self-efficacy implementing PACT for women (DID, 1.0 [0.01,1.94], *p* < 0.05) and higher gender sensitivity (DID, 0.35 [0.02,0.70]; *p* < 0.01). In contrast, in WH-PACTs, EBQI improved clinic leadership change-readiness (DID, 0.80 [0.26,1.34], *p* < 0.01), team-based communication (DID, 0.60 [0.04,1.17], *p* < 0.05), and team functioning (DID, 0.55 [0.10,1.01], *p* < 0.05). At 24 months, EBQI was also associated with significantly lower burnout in WH-PACTs vs. PC-PACTs, when we adjusted for individual-level covariates (adjusted OR, 0.324 [0.11–0.94], *p* = 0.038).

## DISCUSSION

We found that EBQI had mixed impacts on WH care readiness and marginal improvements in self-efficacy in implementing PACT for women and PACT team functioning. However, significant and distinctive impacts of EBQI were seen when we examined effects among those already working in WH-PACTs vs. those working in PC-PACTs. In particular, the lower confidence in providing WH care reflected perceptions of clinicians and staff in PC-PACTs, not those in WH-PACTs. In PC-PACTs, EBQI significantly improved self-efficacy in implementation of PACT for women and gender sensitivity. In contrast, clinic leadership change-readiness, team-based communication, and team function significantly improved in WH-PACTs, with significantly lower burnout at 24 months. EBQI did not have significant effects on team-based decision-making, barriers to comprehensive care for women, or PACT-related QI activities.

We were initially surprised by the finding that EBQI was associated with *lower* confidence in delivering WH care. However, none of the QI roadmaps or local QI teams focused on confidence as a problem, especially since the majority of women Veterans at EBQI VAMCs were seen in WH-PACTs (two QI projects even focused on shepherding women patients to them). WH-PACTs are led by designated WH-PCPs, a designation associated with higher quality and better patient experience among women Veterans, which may have further contributed to the lack of emphasis on confidence.^2,22^ Local dissemination of EBQI activities PACT-wide may have also contributed to a better appreciation of knowledge deficits in WH care, thereby lowering confidence. Unadjusted comparisons demonstrated a significant *increase* in confidence at control VAMCs, which may have had more recent growth in the number of providers trained in the VA OWH’s WH “mini-residency” program (OWH has trained over 10,000 PCPs and nurses since the study’s inception (S. Haskell, personal communication)).^23,24^ Future research should explore ramifications of shifts in confidence for the PC workforce (e.g., cross-coverage issues when WH-PACTs are understaffed).

Like our findings around clinician confidence, overall, EBQI effects on gender sensitivity appear driven by PC-PACT rather than WH-PACT clinicians/staff. This improvement among PC-PACT clinicians/staff is important, as lack of gender sensitivity in VA has been associated with women Veterans’ delaying or forgoing care^25^ or care discontinuity (i.e., no return to primary care 3 years after initial visit).^26^ WH training and experience in working with other WH care professionals are strongly correlated with gender sensitivity, so EBQI may serve as an adjunct to routine training.^27^ Our findings regarding increased gender sensitivity are also consistent with the work of Fox et al., who found that EBQI enhanced gender sensitivity and knowledge in a study of cultural competence training for VA staff.^28^

While not significant overall, we also found that self-efficacy around implementing PACT for women improved only among PC-PACT clinicians/staff. For WH-PACTs, lack of improvement may be due to higher self-efficacy at the outset, fostered by more experience with women Veteran patients and more alignment with national VA WH care policy. PC-PACT clinicians/staff were likely less aware of this policy and may have felt less responsible for its implementation prior to EBQI. Since local QI teams were encouraged to broadly engage PACTs, EBQI may have increased awareness of what it takes to implement PACT for women due to fuller realization of its scope.

Significant improvements in perceived clinic leadership change-readiness and team-based communication among clinicians/staff in WH-PACTs (but not PC-PACTs) may be explained by WH leadership of local QI teams, which may have strengthened team-based communication and QI engagement.^29^ These leaders, in turn, involved an array of local personnel in their local QI projects, sharing knowledge and increasing collaborative problem-solving. Improvements in PACT team function in WH-PACTs may have a similar explanation in addition to the fact that two of the eight EBQI sites focused their QI projects explicitly on improving team function (e.g., virtual huddles).^30^ These improvements may also be associated with unmeasured characteristics of effective team functioning (e.g., shared goals and sense of purpose),^31^ which may be markers of more mature, fully realized PACTs.

Contrary to our expectation, burnout at 24 months was not lower at EBQI vs. control VAMCs. Our previous work suggests that EBQI reduces burnout, possibly by increasing empowerment of local QI teams, but this impact may take more time than the duration of this trial.^15^ However, we did find significantly lower burnout in WH-PACTs, where EBQI was concentrated and where job satisfaction may have been better with the focus on WH care delivery.^32^ Nationally, WH-PCPs have higher rates of burnout compared to general PCPs.^9^ While we lack baseline data enabling us to assess change over time, the differences between WH-PACTs and PC-PACTs is nonetheless notable. Given the crisis of burnout among VA providers more broadly,^33,34^ more attention should be devoted to spreading organizational strategies that successfully combat burnout, such as VA’s recent Reduce Employee Burnout and Optimize Organizational Thriving (REBOOT) national initiative.

Given EBQI’s focus on supporting local QI teams, we hypothesized that EBQI would improve team-based decision-making and PACT-related QI activities and simultaneously reduce barriers to implementation of PACT for women. We found no significant EBQI impacts in these measures. From PACT teamlet interviews, we learned that insufficient staffing, reliance on parttime providers, and access priorities, among other challenges, complicated PC delivery for women Veterans.^8^ These and other barriers we asked about (e.g., inadequate space, limited chaperones) may have been beyond the purview of local QI teams. Projects chosen by local QI teams also involved more people outside of PACT than within (e.g., improve mammography coordination), requiring less within-teamlet decision-making and engagement in QI among teamlet members.

This trial had several limitations. Low response rates—not uncommon among busy PC employees—are concerning; weighting for non-response may not have adequately addressed potential biases. We also lacked burnout measures at baseline, precluding adjustment for any baseline differences that may have been present. This trial also spanned VA’s “access crisis”^35^; some EBQI projects may have been adversely affected (e.g., due to divided attention and heightened organizational demands). We also relied on an unbalanced design to accommodate local variations in how EBQI might be deployed. However, these designs have lower statistical power to detect differences; significant findings demonstrate EBQI’s robustness. Outcomes may have benefited from working in the context of a PBRN where research-clinical dialogue may be better.^36^

In conclusion, we found EBQI to have mixed results overall, but with important and distinctive impacts for different PACT types (WH-PACTs vs. PC-PACTs). Specifically, EBQI increased gender sensitivity and self-efficacy in PC-PACTs, and improved clinic leadership change-readiness, team-based communication, and team functioning in WH-PACTs, with lower burnout at 24 months as well.

Within 1 year of launching EBQI, participating VAMCs began reporting early quality gains from their local QI projects to leadership, several of which were spread VISN-wide. These efforts led the VA OWH to adopt EBQI for use in low-performing VAs^37^ and OWH has since adopted EBQI for national rollout to continue the journey to tailor VA care to women Veterans’ needs, while we study impacts of EBQI on implementation of WH-focused evidence-based practices.

### Supplementary Information

Below is the link to the electronic supplementary material.

Supplementary file1 (DOCX 51.4 KB)

## Data Availability

The datasets generated and analyzed during the current study are not publicly available due to inclusion of potentially sensitive disclosures, potential for subject re-identification, and lack of consent for data sharing from study participants.
