# Supplementary material for: Impact of Evidence-Based Quality Improvement on Tailoring VA’s Patient-Centered Medical Home Model to Women Veterans’ Needs
Source: J Gen Intern Med. 2024 Feb 29;39(8):1349–59. doi: 10.1007/s11606-024-08647-4 (PMC11169220; doi:10.1007/s11606-024-08647-4)
Supplement: Supplementary file 1 — Supplementary file1 (DOCX 51.4 KB) [file 11606_2024_8647_MOESM1_ESM.docx]

Supplemental Appendix 1. PACT Survey Items and Scale

CONFIDENCE PROVIDING CARE FOR WOMEN (k=9, Cronbach’s alpha = 0.93)

How confident are you in your ability to provide each of the following services for women patients at your VA?

|  | Not at all Confident | Somewhat Confident | Moderately Confident | Very Confident |
| --- | --- | --- | --- | --- |
|  | Check one box on each line | | | |
| Conducting well-woman exams (e.g., Pap smears) | □ | □ | □ | □ |
| Screening and/or treatment for sexually transmitted diseases | □ | □ | □ | □ |
| Contraception counseling | □ | □ | □ | □ |
| Evaluation and/or management of breast symptoms  (e.g., mass, fibrocystic breast disease) | □ | □ | □ | □ |
| Evaluation and/or management of acute and chronic pelvic pain | □ | □ | □ | □ |
| Screening for interpersonal and domestic violence | □ | □ | □ | □ |
| Screening for military sexual trauma | □ | □ | □ | □ |
| Addressing service-related injuries or trauma | □ | □ | □ | □ |
| Menopause symptom management (e.g., hormone replacement) | □ | □ | □ | □ |

**SELF-EFFICACY IMPLEMENTING PACT FOR WOMEN PATIENTS (k=6, Cronbach’s alpha = 0.81)**

Please indicate how much you agree or disagree with the following statements about the *implementation* of PACT for women patients.

|  | Strongly disagree | Disagree | Neither disagree nor agree | Agree | Strongly agree |
| --- | --- | --- | --- | --- | --- |
|  | Check one box on each line | | | | |
| As we implement PACT for women patients, I feel I can handle my role with ease. | □ | □ | □ | □ | □ |
| There are some tasks related to PACT for women patients that I should be doing, but don't think I can do well. | □ | □ | □ | □ | □ |
| I have the skills that are needed to make my role in PACT for women patients successful. | □ | □ | □ | □ | □ |
| There are some tasks expected of my role in PACT for women patients that I don’t have time for. | □ | □ | □ | □ | □ |
| My past experiences make me confident that I will be able to perform successfully as changes are made related to PACT for women patients. | □ | □ | □ | □ | □ |
| Changes related to PACT for women patients have been helpful for improving care for women patients. | □ | □ | □ | □ | □ |

PERCEIVED BARRIERS TO PROVIDING COMPREHENSIVE PRIMARY CARE FOR WOMEN PATIENTS (k=15, Cronbach’s alpha = 0.91)

The following items are factors that could limit comprehensive primary care for women patients. In your opinion, how much, if at all, does each factor limit your or your clinic’s ability to provide comprehensive primary care for women patients? If a factor is not present for you or your clinic (e.g., if there *is* sufficient space and structure), check “Does not apply.”

|  | Does not apply | Does not limit | Limits somewhat | Limits a great deal |
| --- | --- | --- | --- | --- |
|  | Check one box on each line | | | |
| Limited female staff available to serve as chaperones for gender-specific exams | □ | □ | □ | □ |
| Limited space and structure (e.g., small rooms, too few rooms, poor layout) | □ | □ | □ | □ |
| Women patients are not comfortable with the presence of men in the clinic (e.g., waiting areas) | □ | □ | □ | □ |
| Inadequate time or opportunity for me to maintain proficiency in women’s health care | □ | □ | □ | □ |
| Inadequate visit time to address physical health issues | □ | □ | □ | □ |
| Inadequate training to address women’s physical health issues (e.g., reproductive health) | □ | □ | □ | □ |
| Inadequate visit time to address mental health issues | □ | □ | □ | □ |
| Inadequate training to address mental health of women patients | □ | □ | □ | □ |
| Discomfort dealing with mental health in women patients (e.g., sexual trauma, PTSD) | □ | □ | □ | □ |
| Teamlets are not adequately staffed | □ | □ | □ | □ |
| Difficulty maintaining teamlet cohesiveness due to staff turnover, or sharing staff across multiple teamlets | □ | □ | □ | □ |
| Difficulty coordinating care with other providers or clinics | □ | □ | □ | □ |
| Lack of support from the local clinical leadership | □ | □ | □ | □ |
| Inadequate training to address health issues of lesbian, gay, bisexual, and transgender patients. | □ | □ | □ | □ |
| Lack of support from direct supervisors within the clinic | □ | □ | □ | □ |
| Other limiting factors (Specify) __________________________ | □ | □ | □ | □ |

GENDER SENSITIVITY (k=10, Cronbach’s alpha = 0.80)

For each statement, choose the answer that best matches your opinion.

|  | Strongly disagree | Disagree | Neither disagree nor agree | Agree | Strongly agree |
| --- | --- | --- | --- | --- | --- |
|  | Check one box on each line | | | | |
| Having female patients at VA primary care clinics makes things too difficult. | □ | □ | □ | □ | □ |
| The VA should not be expected to provide special health services for women. | □ | □ | □ | □ | □ |
| It is nice to have female patients at VA primary clinics. | □ | □ | □ | □ | □ |
| Sometimes I wish VA primary care clinics had only male patients. | □ | □ | □ | □ | □ |
| Special women’s clinics should be at all VA health facilities. | □ | □ | □ | □ | □ |
| It would bother me to see a woman breast feed in the clinic. | □ | □ | □ | □ | □ |
| Female patients care too much about the way the clinic looks. | □ | □ | □ | □ | □ |
| Compared to men, women expect too much courtesy from clinic staff. | □ | □ | □ | □ | □ |
| Having a special room for women to breastfeed would be a good clinic policy. | □ | □ | □ | □ | □ |
| Having female patients makes this a better clinic. | □ | □ | □ | □ | □ |

CLINIC LEADERSHIP CHANGE-READINESS (k=6, Cronbach’s alpha = 0.93)

Please indicate how much you agree or disagree with each of the following statements about the leadership at the primary care or women’s health clinic in which you spend the most time with women patients. If you do not care for women patients, please answer about the primary care clinic where you spend the most time.

|  | Strongly disagree | Disagree | Neither disagree nor agree | Agree | Strongly agree |
| --- | --- | --- | --- | --- | --- |
| **Clinic leadership…** | Check one box on each line | | | | |
| Provides measurable objectives for implementing the strategy and vision within our clinic. | □ | □ | □ | □ | □ |
| Recognizes and rewards progress in implementing change with our clinic. | □ | □ | □ | □ | □ |
| Encourages and supports changes in clinic patterns to improve patient care. | □ | □ | □ | □ | □ |
| Is willing to try new clinical protocols. | □ | □ | □ | □ | □ |
| Works cooperatively with senior leadership/clinical management to make appropriate changes. | □ | □ | □ | □ | □ |
| Understands the difficulties and challenges related to the implementation of PACT. | □ | □ | □ | □ | □ |

TEAM COMMUNICATION (k=5, Cronbach’s alpha = 0.79)

Please indicate how much you agree or disagree with the following statements about your PACT teamlet for women patients. If you do not care for women patients, please answer about the PACT teamlet where you spend the most time.

|  | | **Strongly Disagree** | **Disagree** | **Neither disagree nor agree** | **Agree** | **Strongly agree** |
| --- | --- | --- | --- | --- | --- | --- |
|  |  | Check one box on each line | | | | |
| - - 1. Members of our PACT teamlet for women patients actively share their special knowledge and expertise with one another. |  | 🞎 | 🞎 | 🞎 | 🞎 | 🞎 |
| - - 1. Some members of this PACT teamlet for women patients lack the knowledge and skills that they need to do their parts of the teamlet’s work. |  | 🞎 | 🞎 | 🞎 | 🞎 | 🞎 |
| - - 1. Members of this PACT teamlet for women patients have more than enough talent and experience for the kind of work that we do. |  | 🞎 | 🞎 | 🞎 | 🞎 | 🞎 |
| - - 1. Our PACT teamlet for women patients is quite skilled at capturing the lessons that can be learned from our work experiences. |  | 🞎 | 🞎 | 🞎 | 🞎 | 🞎 |
| - - 1. How seriously a member’s ideas are taken by others on our PACT teamlet for women patients often depends more on who the person is than on how much he or she actually knows. |  | 🞎 | 🞎 | 🞎 | 🞎 | 🞎 |

**TEAM DECISION-MAKING (k=6, Cronbach’s alpha = 0.86)**

Please indicate how much you agree or disagree with each of the following statements about the primary care or women’s health clinic in which you spend the most time with women patients. If you do not care for women patients, please answer about the primary care clinic where you spend the most time.

|  | Strongly disagree | Disagree | Neither disagree nor agree | Agree | Strongly agree |
| --- | --- | --- | --- | --- | --- |
|  | Check one box on each line | | | | |
| Staff and clinicians are involved in developing plans for improving quality. | □ | □ | □ | □ | □ |
| In this clinic, when I have a conflict with a co-worker from a different clinical or administrative discipline, I can access help to resolve the problem. | □ | □ | □ | □ | □ |
| This clinic encourages staff and clinicians’ input for making changes and improvements. | □ | □ | □ | □ | □ |
| This clinic defines success as teamwork and concern for people. | □ | □ | □ | □ | □ |
| All of the staff and clinicians participate in important decisions about clinical operations (e.g., workflow). | □ | □ | □ | □ | □ |
| In this clinic, co-workers from different clinical or administrative backgrounds frequently interact to solve quality of care problems. | □ | □ | □ | □ | □ |
| Our staff and clinicians have constructive work relationships. | □ | □ | □ | □ | □ |
| There is often tension between people in this clinic. | □ | □ | □ | □ | □ |
| The staff and clinicians in this clinic operate as real teams. | □ | □ | □ | □ | □ |

**HELPFULNESS OF PACT QI ACTIVITIES (k=10, Cronbach’s alpha = 0.80)**

Please answer “Yes” or “No” to the questions below about your exposure to various activities over the past two years. If you answer “Yes” to a question, also rate how helpful you have found that activity for improving patient care.

|  |  | **IF YES:  How helpful is this activity?** | | |
| --- | --- | --- | --- | --- |
|  |  | Not at all helpful | Somewhat helpful | Very helpful |
| Over the past two years… | Check one | Check one | | |
| Have you used measurement tools to help assess your performance? | - Yes **⇨** - No - Don’t know |  |  |  |
| Has your clinic used information systems (e.g., PACT Compass, PCMM) to provide timely data and feedback to staff on patient utilization outcomes and experiences? | - Yes **⇨** - No - Don’t know |  |  |  |
| Have you received regular reports or feedback on your performance? | - Yes **⇨** - No - Don’t know |  |  |  |
| Did your clinic use centralized clerks for scheduling appointments? | - Yes **⇨** - No - Don’t know |  |  |  |
| Were you involved in any small tests of change to improve quality of care (e.g., testing out new patient care approaches on a few patients or providers first)? | - Yes **⇨** - No - Don’t know |  |  |  |

**PACT TEAM FUNCTION (k=4, Cronbach’s alpha = 0.88)**

Please indicate how much you agree or disagree with each of the following statements about the PACT teamlet in which you spend the most time with women patients. If you do not care for women patients, please answer about the PACT teamlet where you spend the most time.

|  | Strongly disagree | Disagree | Neither disagree nor agree | Agree | Strongly agree |
| --- | --- | --- | --- | --- | --- |
|  | Check one box on each line | | | | |
| Everyone in this teamlet has the special skills that are needed for teamlet work. | □ | □ | □ | □ | □ |
| Overall, I am satisfied with how my teamlet members work together. | □ | □ | □ | □ | □ |
| My core teamlet members (e.g., RN, LPN, or clerk) are available (in person or by phone/messaging) whenever I need assistance during face-to-face patient encounters | □ | □ | □ | □ | □ |
| The core teamlet members (e.g., RN, LPN, or clerk) are available (in person or by phone/messaging) whenever assistance is needed with administrative patient care activities (e.g., charting, entering orders) | □ | □ | □ | □ | □ |

PRIMARY CARE BURNOUT (k=1)

How often does the following statement apply to you: “I feel burned out from my work”?

Check one.

- Never
- A few times a year
- Every month
- A few times a month
- Every week
- A few times a week
- Every day

**Appendix Table 2: Unadjusted Comparisons of Outcome Measures**

**Between PC-PACTs and WH-PACTs**

| **Outcome Measures** | **Baseline** | | | | | **24-month** | | | | |
| --- | --- | --- | --- | --- | --- | --- | --- | --- | --- | --- |
|  |  |  |  |  |  |  |  |  |  |  |
|  | **PC-PACTs** | | **WH-PACTs** | |  | **PC-PACTs** | | **WH-PACTs** | |  |
|  | **n** | **mean** | **n** | **Mean** | **p-value** | **n** | **Mean** | **n** | **Mean** | **p-value** |
| **Women’s Health Care Readiness** | | | | | | | | | | |
| **Confidence in delivering WH care*** (mean score on 4-point scale, 1=not at all, 2=somewhat, 3=moderately, 4=very confident) | 49 | 2.39 | 38 | 3.23 | <0.001 | 43 | 2.30 | 41 | 3.43 | <0.001 |
| **Self-efficacy for implementing PACT for women** (mean score on 5-point agreement scale, 1=strongly disagree to 5=strongly agree) | 15 | 3.23 | 105 | 3.68 | 0.17 | 88 | 3.22 | 111 | 3.91 | 0.61 |
| **Barriers to WH PACT implementation** (mean score 3-point scale, 1=does not limit, 2=limits somewhat, 3=limits a great deal) | 149 | 1.74 | 106 | 1.74 | 0.97 | 89 | 1.55 | 111 | 1.43 | 0.12 |
| **Gender sensitivity** (mean score on 5-point agreement scale, 1=strongly disagree to 5=strongly agree) | 154 | 3.97 | 106 | 4.26 | <0.001 | 90 | 3.95 | 111 | 4.33 | <0.001 |
| **Team-Based Primary Care** | | | | | | | | | | |
| **Clinic leadership change-readiness** (mean score on 5-point agreement scale, 1=strongly disagree to 5=strongly agree) | 145 | 3.23 | 104 | 3.35 | 0.37 | 88 | 3.45 | 111 | 3.59 | 0.35 |
| **Team communication** (mean score on 5-point agreement scale, 1=strongly disagree to 5=strongly agree) | 147 | 3.43 | 103 | 3.42 | 0.87 | 89 | 3.58 | 111 | 3.76 | 0.12 |
| **Team-based decision making** (mean score on 5-point agreement scale, 1=strongly disagree to 5=strongly agree) | 147 | 3.44 | 103 | 3.53 | 0.44 | 89 | 3.69 | 111 | 3.81 | 0.33 |
| **Helpfulness of PACT QI activities** (mean score on 3-point scale, 1=not at all helpful, 2=somewhat helpful, 3=very helpful) | 152 | 1.89 | 105 | 1.78 | 0.15 | 84 | 2.21 | 106 | 2.33 | 0.14 |
| **PACT team functioning** (mean score on 5-point agreement scale, 1=strongly disagree to 5=strongly agree) | 18 | 3.61 | 107 | 3.85 | 0.32 | 86 | 3.75 | 109 | 4.01 | 0.036 |

Negatively worded survey items were reverse coded, high scores reflect high attribute (e.g., high confidence, high barriers, high function). Weighted for non-response.

^*^The items for this measure were asked among clinicians only and not staff.
